# Bioinspired and Biomimetic Nanotherapies for the Treatment of Infectious Diseases

**DOI:** 10.3389/fphar.2019.00751

**Published:** 2019-07-05

**Authors:** Guoyu Yang, Sheng Chen, Jianxiang Zhang

**Affiliations:** ^1^Department of Pharmaceutics, College of Pharmacy, Third Military Medical University, Chongqing, China; ^2^The First Clinical College, Chongqing Medical University, Chongqing, China; ^3^Department of Pediatrics, Southwest Hospital, Third Military Medical University, Chongqing, China

**Keywords:** biomimetic, bioengineering, nanoparticle, targeted therapy, vaccine, infectious disease

## Abstract

There are still great challenges for the effective treatment of infectious diseases, although considerable achievement has been made by using antiviral and antimicrobial agents varying from small-molecule drugs, peptides/proteins, to nucleic acids. The nanomedicine approach is emerging as a new strategy capable of overcoming disadvantages of molecular therapeutics and amplifying their anti-infective activities, by localized delivery to infection sites, reducing off-target effects, and/or attenuating resistance development. Nanotechnology, in combination with bioinspired and biomimetic approaches, affords additional functions to nanoparticles derived from synthetic materials. Herein, we aim to provide a state-of-the-art review on recent progress in biomimetic and bioengineered nanotherapies for the treatment of infectious disease. Different biomimetic nanoparticles, derived from viruses, bacteria, and mammalian cells, are first described, with respect to their construction and biophysicochemical properties. Then, the applications of diverse biomimetic nanoparticles in anti-infective therapy are introduced, either by their intrinsic activity or by loading and site-specifically delivering various molecular drugs. Bioinspired and biomimetic nanovaccines for prevention and/or therapy of infectious diseases are also highlighted. At the end, major translation issues and future directions of this field are discussed.

## Introduction

Infectious diseases caused by pathogenic organisms such as viruses, bacteria, fungi, and parasites are still responsible for the majority of hospitalization and death worldwide. Although considerable achievement has been made by using antibiotic agents varying from small-molecule drugs, peptides/proteins, to nucleic acids, there remain great challenges in effective treatment of infectious diseases (Metcalf and Lessler, 2017; De Rycker et al., 2018; Kaufmann et al., 2018). In addition to severe side effects of currently existing antiviral and antibacterial agents due to their systemic exposure, the rapid emergence of drug resistance is a serious global health problem (Baker et al., 2018; Meylan et al., 2018). Moreover, new viruses and bacteria are constantly emerging by evolution or other biological events, leading to a continuing challenge in control and prevention of infectious diseases. Consequently, in addition to discovering new antiviral agents and antibiotics for infection control, creative strategies need to be developed to maximize efficacy of currently available drugs (Willing et al., 2011; Dickey et al., 2017). Indeed, as one of the most straightforward and cost-effective strategies, vaccination has greatly reduced the morbidity and mortality resulting from infectious diseases. Unfortunately, vaccines remain unavailable for many infectious diseases (Haque et al., 2018), such as *Chlamydia* and *M. tuberculosis* infections. In other cases, there are still safety concerns for the use of vaccines based on inactivated or attenuated live pathogens, while recently developed subunit vaccines usually show poor immunogenicity. Accordingly, innovative technologies are desperately needed for vaccine development (Pardi et al., 2018).

Nanotechnology has been emerging as a new strategy to circumvent multiple disadvantages of antiviral drugs and antibiotics as well as to potentiate their therapeutic benefits (Hallaj-Nezhadi and Hassan, 2015; Milovanovic et al., 2017; Singh et al., 2017). In this aspect, a diverse array of nanoparticles have been examined to improve the efficacy and decrease side effects of various therapeutics in the treatment of infectious diseases (Xiong et al., 2014), by increasing drug solubility/stability, prolonging circulation time, overcoming biological barriers, enhancing bioavailability, targeting infection sites, and modulating drug release profiles in response to biochemical signals relevant to pathological changes (Zaidi et al., 2017; Gao et al., 2018; Zhou et al., 2018). Furthermore, increasing evidence has demonstrated that nanoparticle-based strategies hold great potential to reduce resistance development or even reverse acquired resistance (Kumar et al., 2018), by promoting intracellular uptake, changing the delivery route in subcellular organelles, modulating drug–pathogen interactions, and/or conferring anti-biofilm effect (Abed and Couvreur, 2014; Forier et al., 2014; Shimanovich and Gedanken, 2016; Han et al., 2017).

In addition, numerous studies have substantiated that nanoparticles are promising for amplifying the activity of antigens and adjuvants by promoting humoral and cellular immunity (Bachmann and Jennings, 2010; Swartz et al., 2012; Stewart and Keselowsky, 2017). Due to their unique biophysicochemical properties, nanoparticles can protect antigens from proteolytic degradation and achieve targeted delivery in immunity-related lymphoid tissues, antigen-presenting cells, and subcellular compartments of interest (Lin et al., 2018; Pati et al., 2018; Al-Halifa et al., 2019). Based on these features, nanoparticles allow lower doses of subunit vaccines to afford desirable immune responses, and notably inhibit the nonspecific immune activation resulting from systemic delivery of soluble antigens (Xu et al., 2016; Bookstaver et al., 2018; Lybaert et al., 2018). Also, antigens can be site-specifically delivered to mucosal surfaces, thereby generating expected mucosal immunity for protection against pathogens transmitted *via* the mucosa (Brito and O’Hagan, 2014; Narasimhan et al., 2016). Furthermore, nanoparticle-mediated intracellular delivery of exogenous antigens is able to notably increase cross-presentation, which is highly preferred for protective and therapeutic vaccines (Smith et al., 2013a).

Recently, nanotechnology has been combined with biomimetic strategies to create bioinspired nanoparticles with optimized surface biophysicochemical properties for drug delivery and vaccine development (Angsantikul et al., 2015; Luk and Zhang, 2015; Parodi et al., 2017; Zhang et al., 2017a; Zhou et al., 2017). These biomimetic nanoparticles possess multiple advantages, such as the diversity, tailorability, and reproducibility of synthetic nanomaterials as well as the functionality, complexity, and biocompatibility of biological materials (Yoo et al., 2011; Meyer et al., 2015). Due to their intrinsic activity, nature-inspired nanoparticles themselves can function as effective nanotherapies or nanovaccines against infectious diseases. Alternatively, they may serve as advanced nanocarriers for site-specific delivery of therapeutics or vaccines. Herein, we review recent progress in biomimetic and bioengineered nanotherapies for the management of infectious diseases. Different nanoparticles based on viruses, bacteria, and mammalian cells are first introduced. We then highlight applications of different biomimetic nanoparticles in anti-infective therapy. Also, nanovaccines engineered from bioinspired strategies are described, with emphasis on the prevention or therapy of infectious diseases. Finally, major issues regarding the translation and clinical applications of these bioinspired nanotherapies are discussed.

## Different Biomimetic Nanoparticles Developed for the Treatment of Infectious Diseases

Over the past decades, nanoparticles derived from viruses, bacteria, and mammalian cells have been developed for delivery of diagnostic or therapeutic agents for the management of infectious diseases. Due to their special properties, these biomimetic nanovehicles can effectively transport the loaded cargo molecules to diseased sites without inducing immune responses. Herein, we briefly introduce different types of biomimetic and bioinspired nanoparticles used for therapy of infectious diseases.

### Virus-Based Nanoparticles

**Figure 1:**
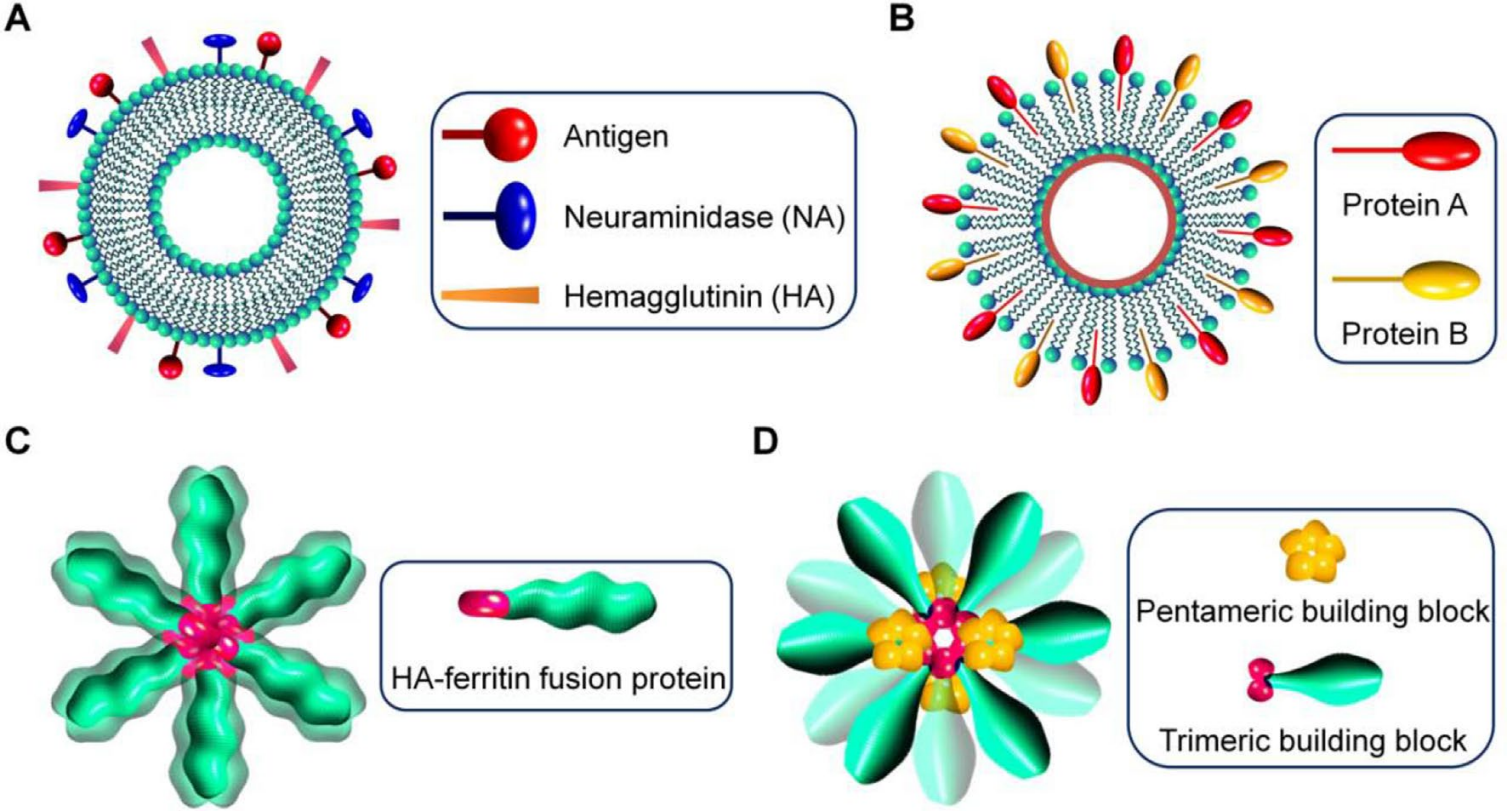
Schematic illustration of virus-mimetic nanoparticles. **(A)** Virosomes. **(B)** Virus-like particles (VLPs). **(C)** Self-assembling nanoparticles displaying antigens on their surface. **(D)** Fully synthetic nanoparticles mimicking viruses.

Viruses can efficiently bind to host cells by specific interactions between virion proteins and lipids, membrane proteins, or carbohydrate moieties on the cell surface. This attachment is generally followed by virus entry in host cells *via* endocytosis/pinocytosis or fusion/penetration. Moreover, viruses have developed various strategies to evade the host immune system. Accordingly, different approaches have been established to construct biomimetic nanoparticles, taking advantage of the unique capabilities of viruses (**Figure 1** and **Table 1**).

**Table 1:** Different biomimetic nanoparticles discussed in this review.

Types of biomimetic nanoparticles		Definition	Applications	References
Virus-based nanoparticles	Virosomes	Spherical vesicles consisting of phospholipid bilayers with incorporated virus-derived proteins.	Vaccination, drug/gene delivery	(Liu et al., 2015; Mohammadzadeh et al., 2016; Blom et al., 2017a; Blom et al., 2017b; Mohammadzadeh et al., 2017; Lederhofer et al., 2018; Li et al., 2018)
	Virus-like particles	Assemblies of viral capsids or envelope proteins derived from viruses.	Vaccines, drug delivery	(Smith et al., 2013a; Smith et al., 2013b; Liu et al., 2016; Collins et al., 2017; Huang et al., 2017; Rohovie et al., 2017; Hill et al., 2018)
	Self-assembling nanoparticles	Nanoparticles self-assembled by natural proteins with displayed viral glycoproteins or other antigens.	Vaccines	(Kanekiyo et al., 2013; Gallagher et al., 2018; Corbett et al., 2019; Rappuoli and Serruto, 2019)
	Fully synthetic nanoparticles	Fully synthesized nanoparticles that can mimic functions of viruses.	Drug delivery, gene therapy	(Marcandalli et al., 2019; Rappuoli and Serruto, 2019)
Nanoparticles derived from bacteria or fungi	Nanoparticles derived from components of the bacterial/fungal membrane	Nanoparticles directly formulated from the components of the bacterial or fungal membrane or nanoparticles based on a chemically modified component of the bacterial or fungal membrane.	Targeted drug delivery	(Tang et al., 2018)
	Outer membrane vesicles	Also referred to as bacterial extracellular vesicles, which are spherical bilayered structures enriched with bioactive molecules, such as proteins and nucleic acids.	Biomimetic therapies, targeted drug delivery	(Watanabe, 2016; Ahmadi Badi et al., 2017; Liu et al., 2017; Liu et al., 2018b; Schulz et al., 2018; Wang et al., 2018a; Wang et al., 2018b; Yu et al., 2018; Wang et al., 2019)
	Nanoparticles coated with bacterial membrane	Synthetic nanoparticles decorated with the bacterial-derived membrane	Antibacterial nanotherapies, vaccines	(Gao and Zhang, 2015)
Nanoparticles mimicking mammalian cells	Cell membrane-coated nanoparticles	Synthetic nanoparticles coated with different cell membranes derived from mammalian cells, such as red blood cells, platelets, leukocytes, dendritic cells, and stem cells.	Biodetoxification, drug/gene delivery, vaccines	(Hu et al., 2011; Parodi et al., 2012; Hu et al., 2013a; Fang et al., 2015b; Hu et al., 2015; Rao et al., 2017; Chen et al., 2018; Fang et al., 2018; Molinaro et al., 2018; Chen et al., 2019)
	Nanoparticles decorated with functional biomolecules	Nanoparticles decorated with plasma membrane-derived proteins, simultaneously coated with multiple biomolecules, or coated with biomimetic molecules to mimic the natural process of different mammalian cells.	Targeted drug/gene delivery	(Robbins et al., 2010; Rodriguez et al., 2013; Molinaro et al., 2016)
	Shedding microvesicles	Vesicles formed by the outward budding of the cytoplasmic membrane.	Targeted drug delivery	(Cocucci et al., 2009; Fuhrmann et al., 2015; Van Niel et al., 2018)
	Exosomes	Extracellular vesicles originated from exocytosis of multivesicular bodies by eukaryotic cells.	Nanotherapies, targeted drug/gene delivery	(Sun et al., 2010; Nolte-’t Hoen and Wauben, 2012; Rani et al., 2015; Gao et al., 2016; Gao et al., 2017; Jansen et al., 2017; Yurkin and Wang, 2017)
Other biomimetic nanoparticles	High-density lipoproteins	Lipid-protein nanoparticles with a mean diameter of 8–10 nm, carrying a large number of different proteins such as apolipoproteins.	Biomimetic nanotherapies, drug delivery, biodetoxification	(Wu et al., 2004; Bricarello et al., 2010; Ma et al., 2018; Raut et al., 2018)

In the first strategy, virus-derived proteins are incorporated in unilamellar liposomes that are spherical vesicles consisting of phospholipid bilayers, resulting in the formation of virosomes with size ranging from 20 to 200 nm (Almeida et al., 1975; Kaneda, 2000) (**Figure 1A**). Generally, envelope glycoproteins derived from influenza virus, such as hemagglutinin (HA) and neuraminidase (NA), are reconstituted with liposomes to prepare virosomes for vaccination or delivery of different therapeutics (Daemen et al., 2005). Also, other enveloped viruses, such as hemagglutinating virus of Japan (HVJ), respiratory syncytial virus (RSV), and vesicular stomatitis virus (VSV), can be used to generate virosomes (Stegmann et al., 2010; Liu et al., 2015; Mohammadzadeh et al., 2016; Mohammadzadeh et al., 2017; Lederhofer et al., 2018). In other cases, human hepatitis B virus-derived nanoparticles were fused with liposomes, giving rise to virosome-like particles (Kaneda, 2012). In addition to affording structural stability to virosomes, the introduced lipoproteins are responsible for disease targeting, cellular uptake, and endolysosomal escape after internalization (De Jonge et al., 2006). Virosomes have multiple advantages, such as ease of production and modification, biodegradability, biocompatibility, promoted fusion activity in the endolysosomes, and capabilities of delivering various drugs and protecting biologics from degradation. Nevertheless, their broad applications remain limited, largely due to the potential risk of immunogenicity. This issue can be partly addressed by modification of virosomes with polyethylene glycol (PEG) and targeting moieties (Khoshnejad et al., 2007; Saga and Kaneda, 2013). In this aspect, different ligands and antibodies are incorporated into virosomes to reduce off-target effects (Khoshnejad et al., 2007; Li et al., 2018).

In the second approach, virus-like particles (VLPs) are assembled by viral capsids or envelope proteins derived from viruses, with the advantages of precisely defined structures, capacity of packaging different drugs, and displaying functional moieties on their surfaces (**Figure 1B**). Of note, VLPs can also be formed by synthetic viral capsids (Matsuura et al., 2010). The pristine VLPs can be further modified to afford additional functions by tailoring VLP proteins *via* genetic and chemical engineering (Smith et al., 2013b; Hill et al., 2018). For instance, hydrophilic polymers are conjugated on VLPs to increase stability, prolong circulation time, reduce nonspecific absorption, or attenuate immune responses (Steinmetz and Manchester, 2009; Manzenrieder et al., 2011). On the other hand, to overcome the disadvantages of the natural tropism of VLPs, different chemical functionalization methods have been developed to conjugate various targeting ligands on VLPs for site-specific drug delivery (Smith et al., 2013b). Since the antigenicity of VLPs is comparable to their parent viruses, they are initially synthesized for vaccination. Nevertheless, VLPs can be engineered to deliver various drugs varying from small-molecule drugs, peptides, proteins, to nucleic acids, in which therapeutic molecules are packaged by either non-covalent interaction-mediated physical loading or chemical conjugation (Wu et al., 2009; Kwak et al., 2010; Rohovie et al., 2017).

**Figure 2:**
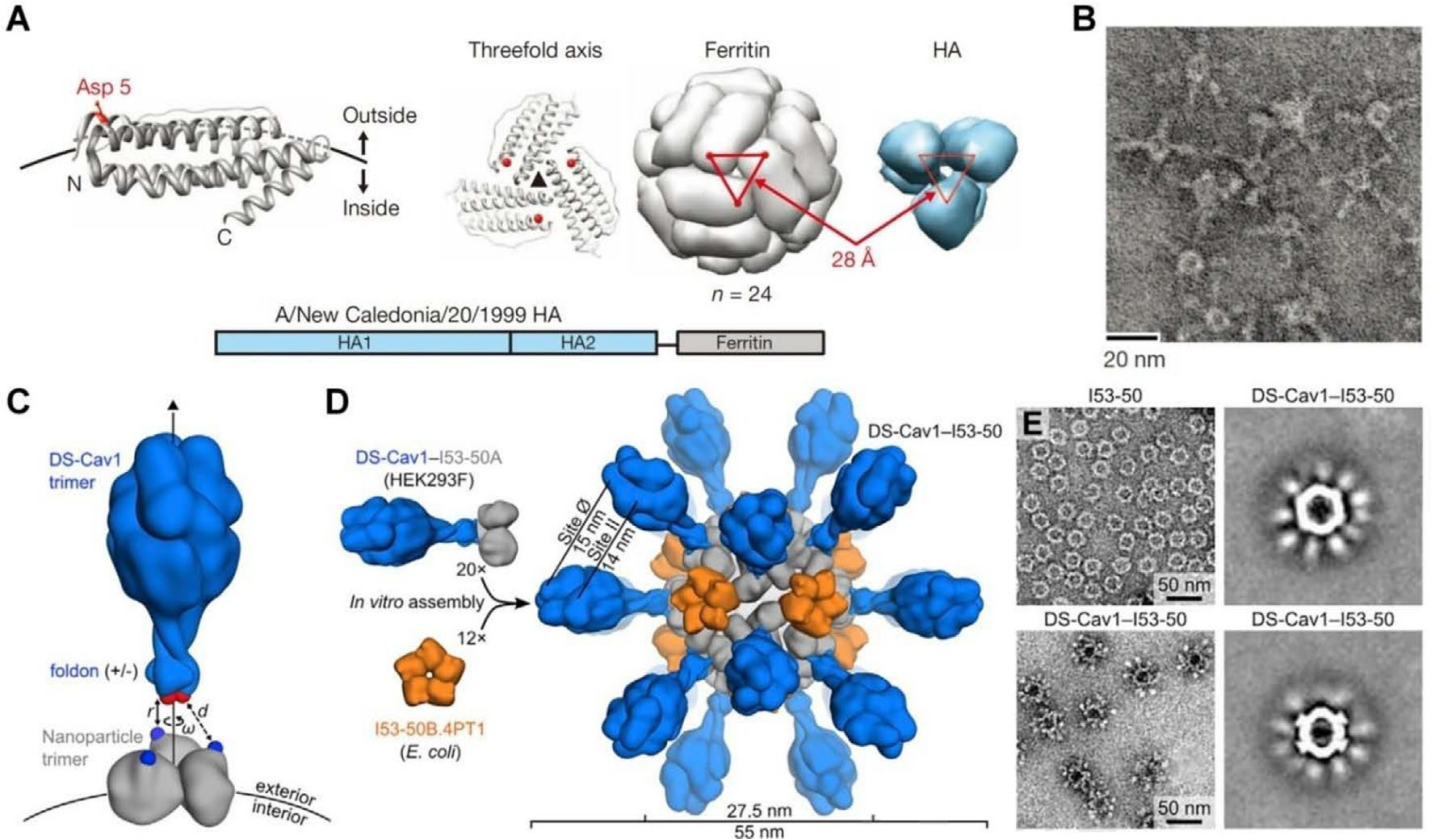
Molecular design and characterization of virus-mimicking self-assembling nanoparticles. **(A, B)** Construction of hemagglutinin (HA)-ferritin fusion protein-assembled nanoparticles displaying influenza virus HA. **(A)** A subunit of *H. pylori* non-haem ferritin [protein data bank (PDB: 3bve)] (left). The NH_2_- and COOH-termini are labeled as N and C, respectively. Three subunits surrounding a threefold axis are shown (middle) and Asp 5 is colored in red. An assembled ferritin nanoparticle and an HA trimer (PDB: 3sm5) (viewed from membrane proximal end) (right). A triangle connecting the Asp 5 residues at the threefold axis is shown in red. The same triangle is drawn on the HA trimer (right). A schematic representation of the HA-ferritin fusion protein is shown (bottom). **(B)** Negatively stained transmission electron microscopy (TEM) images of HA nanoparticles. **(C–E)** Design, *in vitro* assembly, and structural characterization of DS-Cav1-I53-50. **(C)** Schematic representation of the computational docking protocol used to identify nanoparticle components suitable for fusion to DS-Cav1. The C termini of the foldon and N termini of the nanoparticle trimer are shown as red and blue spheres, respectively, and the exterior and interior surfaces of the nanoparticle are depicted. **(D)** Structural model of DS-Cav1-I53-50 and schematic of the *in vitro* assembly process. Each nanoparticle comprises 20 trimeric and 12 pentameric building blocks for a total of 60 copies of each subunit. **(E)** Negatively stained TEM image of I53-50 and DS-Cav1-I53-50 nanoparticles. The two images on the right are averages of negatively stained particles. Images **(A)** and **(B)** are reprinted with permission from Kanekiyo et al. (2013). © (2013) Macmillan Publishers Limited. Images **(C)–(E)** are reprinted with permission from Marcandalli et al. (2019). © (2019) Elsevier Inc.

In a new strategy, self-assembling nanoparticles are obtained by displaying viral glycoproteins with natural proteins that have the ability to form nanoparticles by self-assembly (**Figure 1C**). An elegant example was shown in a study by Nabel and colleagues (Kanekiyo et al., 2013), in which influenza HA was genetically fused to ferritin, and the obtained fusion glycoprotein can spontaneously assemble into nanoparticles, thereby exposing eight HA trimers on their surface (**Figure 2A and B**). Most recently, a computational protein design method was used to develop a self-assembling nanoparticle bearing antigen from RSV (**Figure 1D**) (Marcandalli et al., 2019). In this case, a rationally designed, self-assembling protein nanoparticle serves as a scaffold for multivalent presentation of a prefusion-stabilized variant of the F glycoprotein trimer of RSV, with a repetitive array and controllable density (**Figure 2C–E**). This *in silico* designed and fully synthetic nanoparticle exhibits optimized stability, immunogenicity, and adjuvanticity (Marcandalli et al., 2019).

Finally, virus-mimetic nanoparticles have been developed by different biomimetic methods. Bae et al. synthesized a virus-mimetic nanogel that consists of a hydrophobic polymer core and a capsid-like outer shell (Lee et al., 2008). The obtained nanovehicle can significantly mimic viral properties, since it is able to efficiently infect cells, effectively kill cells, and migrate to neighboring cells. A virus-mimetic nanocapsule was constructed by self-assembly of iron oxide nanoparticles and a one-component functional protein lactoferrin, which can encapsulate both hydrophilic and hydrophobic drugs (Fang et al., 2015a). By mimicking different modular components of viruses, a multicomponent virus-mimetic nanoparticle was prepared, in which membrane-disrupting peptides, nucleic acid binding components, a protective layer, together with an outer targeting ligand were incorporated for programmed gene delivery (Soliman et al., 2012). Recently, a polymer-templated protein nanoball with well-controlled displaying of HA1 on its surface was prepared as a novel influenza virus-mimetic nanoparticle (Lee et al., 2018). Using silica-based nanoparticles, nonviral nanoparticles mimicking virus surface topography were successfully synthesized by Yu and coworkers, which showed enhanced cellular delivery of small interfering RNA (siRNA) (Niu et al., 2013).

### Nanoparticles Derived From Bacterial or Fungal Pathogens

Since recognition interactions among biomolecules on the bacterial/fungal membrane and host cells is the first step toward their adhesion and entry into target cells (Pizarro-Cerda and Cossart, 2006), different components of the bacterial/fungal membrane have been used for preparation of nanoparticles to partly recapitulate their natural features or to realize targeted treatment of bacterial or fungal infection (**Table 1**). For instance, chitosan, an important component of the vegetative cell wall of *C. neoformans* (Baker et al., 2007), was utilized to formulate nanoparticles, resulting in a *C. neoformans*-targeted drug delivery system (Tang et al., 2018).

Another type of bacterial-derived nanoparticles are bacterial extracellular vesicles, also referred to as outer membrane vesicles (OMVs), which are spherical bilayered structures (10–30 nm in diameter) enriched with bioactive molecules, such as proteins and nucleic acids (Kim et al., 2015). OMVs have been investigated as therapies or biomimetic nanovehicles for targeted drug delivery (Watanabe, 2016; Liu et al., 2017; Liu et al., 2018b; Schulz et al., 2018; Wang et al., 2018b; Yu et al., 2018; Wang et al., 2019). Based on the similar consideration, microbiota-derived extracellular vesicles were examined for therapeutic applications (Ahmadi Badi et al., 2017), in view of the fact that dysbiosis of gut microbiota is closely related to the pathogenesis of an astounding array of diseases (Tremaroli and Bäckhed, 2012). Moreover, these vesicles contain numerous types of compounds capable of affecting diverse pathways in the host. Besides, OMVs can be used to prepare functional nanoparticles with coated bacterial membrane for the development of effective antibacterial nanotherapies or nanovaccines (Gao et al., 2015). Generally, OMVs are produced by membrane blebbing or membrane budding, which are then isolated for different applications (Brown et al., 2015). To overcome limitations of OMVs based on this natural process, such as poor batch-to-batch quality control and barriers to scalability, a facile method was established by Wang and coworkers (Wang et al., 2018a). In this approach, bacterial membrane is physically disrupted by nitrogen cavitation. The cellular membrane thus obtained can spontaneously form nanoscale vesicles. It should be noted that, in addition to bacteria, extracellular vesicles may be produced from fungi (Brown et al., 2015).

### Nanoparticles Mimicking Mammalian Cells

**Figure 3:**
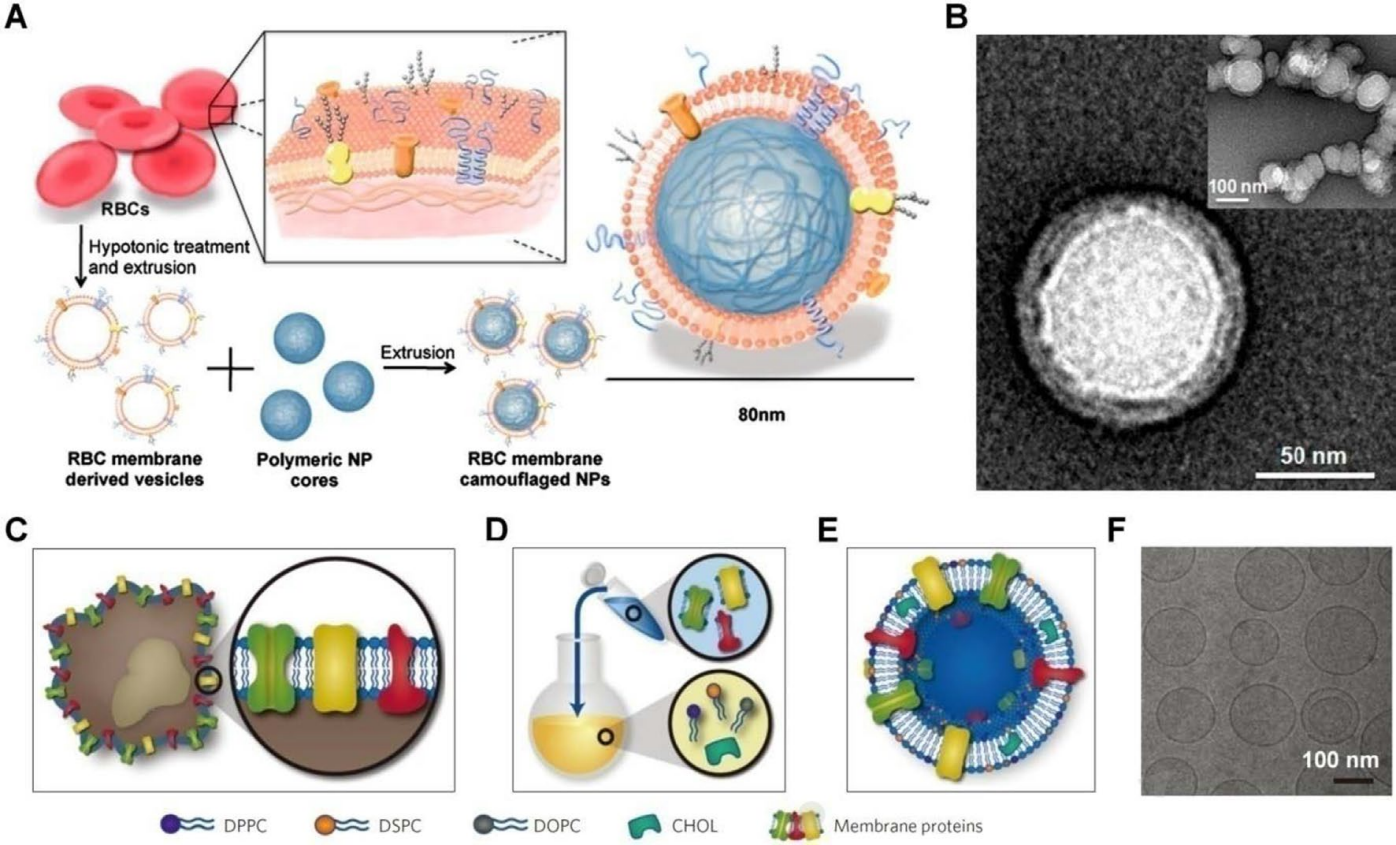
Engineering of cell-mimetic nanoparticles. **(A)** Schematic illustration of the preparation process of the red blood cell (RBC) membrane-coated poly(lactic-co-glycolic acid) (PLGA) nanoparticles. **(B)** Representative TEM images of uranyl acetate-stained RBC membrane-coated PLGA nanoparticles (NPs). **(C–F)** Synthesis, formulation, and characterization of leukosomes. **(C)** Extraction of proteolipid materials from murine J774 macrophages. **(D)** Protein enrichment of the phospholipid film. **(E)** Vesicular formulation of leukosomes. **(F)** Cryo-TEM analysis of leukosomes. NPs, nanoparticles; DPPC, 1,2-dihexadecanoyl-sn-glycero-3-phosphocholine; DSPC, 1,2-distearoyl-sn-glycero-3-phosphocholine; DOPC, 1, 2-dioleoyl-sn-glycero-3-phosphocholine; CHOL, cholesterol. Images **(A)** and **(B)** are reprinted with permission from Hu et al. (2011). © (2011) National Academy of Sciences. Images **(C)–(F)** are reprinted with permission from Molinaro et al. (2016). © (2016) Macmillan Publishers Limited.

Recently, different strategies have been developed to engineer nanoparticles mimicking the diverse functions of various types of mammalian cells, including red blood cells (RBCs), platelets, leukocytes, dendritic cells, and stem cells (**Table 1**) (Parodi et al., 2012; Hu et al., 2013a; Hu et al., 2015; Cheng et al., 2018; Chu et al., 2018; Zhang et al., 2018). Initially, the erythrocyte membrane was coated on poly(lactic-co-glycolic acid) (PLGA) nanoparticles to extend their *in vivo* circulation time (**Figure 3A and B**) (Hu et al., 2011). Since then, cell membranes derived from different cells have been examined for surface functionalization of a variety of nanomaterials, including nanoparticles based on synthetic or natural polymers, silica, gold, and iron oxide, as well as nanogels, quantum dots, upconversion nanoparticles, and metal organic frameworks (Fang et al., 2015b; Fang et al., 2018). To prepare cell membrane-coated nanoparticles, different coating technologies can be used. In most studies, the coextrusion method is adopted, in which nanoparticulate cores and membrane vesicles of interest are extruded through a porous membrane with specific pore size. Alternatively, microfluidic systems can be used to cloak nanoparticles with cellular membranes (Rao et al., 2017; Molinaro et al., 2018).

Nevertheless, scale-up preparation and translation studies of the cell membrane-coated nanoparticles remain limited, largely due to the complicated synthetic routes and purification procedures as well as relatively poor reproducibility. To overcome these limitations, Tasciotti’s group developed a method that can leverage the advantages of bottom-up and top-down strategies (**Figure 3C–F**) (Molinaro et al., 2016; Molinaro et al., 2018). In this scenario, proteins derived from the leukocyte plasma membrane were incorporated into lipid nanoparticles, resulting in leukosomes capable of encapsulating drugs with variable solubility and site-specifically delivering the cargo molecules to the inflamed endothelium.

In addition, nanoparticles have been decorated with functional molecules to achieve specific biological features. For example, leukocyte-like avid adhesion was achieved by nanoparticles coated with ligands that mediate two leukocyte adhesion pathways (Robbins et al., 2010). In this case, polymersomes were functionalized with sialyl Lewis X and an intercellular adhesion molecule (ICAM)-1 antibody, resulting in avid and selective binding to surfaces coated with P-selectin and ICAM-1 (both are adhesion molecules involved in inflammatory diseases), even under flow. On the other hand, macrophage-mediated clearance of intravenously delivered nanoparticles can be significantly delayed by coating with self peptides that are computationally designed from a human membrane protein CD47 (Rodriguez et al., 2013).

Also, mammalian cell-derived vesicles (CDVs) have been extensively explored for their intrinsic activities and diversity in therapeutic loading capacity (Fuhrmann et al., 2015; Van Niel et al., 2018). Shedding microvesicles and exosomes are two major types of CDVs, which can be generated by almost all cell types in physiological and pathological conditions. Shedding microvesicles (with diameter of 10–1,000 nm) are formed by the outward budding of the cytoplasmic membrane, while exosomes (30–200 nm) originate from exocytosis of multivesicular bodies (Cocucci et al., 2009). Because CDVs are important mediators responsible for intercellular communications and the horizontal transfer of membrane/cargo molecules (such as cytokines, proteins, and nucleic acids), they have been investigated as modulators, regulators, diagnostic probes, and therapeutic targets (Nolte-’t Hoen and Wauben, 2012; Rani et al., 2015; Jansen et al., 2017). Taking advantage of their innate capacities to escape immune clearance, recognize target cells, and overcome biological barriers, CDVs are also used as smart drug delivery carriers, either in their native forms or after re-engineering by genetic or chemical modification (Ha et al., 2016; Vader et al., 2016; Armstrong et al., 2017).

Another type of CDVs can be directly formulated by mammalian cells. As an elegant example, Wang’s group has established a nitrogen cavitation technique to efficiently generate cell membrane nanovesicles (Gao et al., 2016; Gao et al., 2017; Yurkin and Wang, 2017). In this approach, mechanical forces produced by nitrogen cavitation can rapidly disrupt cell membranes of activated neutrophils, and subsequent assembly of the broken membrane gives rise to nanoscale vesicles, with thickness and protein profiles similar to those of the cell membrane of parent cells. Compared to other methods used for the preparation of CDVs, this technique shows high yield, good reproducibility, and desirable scale-up capacity.

### Other Biomimetic Nanoplatforms

Besides the above-mentioned nanoparticles, other biomimetic nanoplatforms, such as hepatitis B virus envelope L particles and lipid nanoparticles (**Table 1**), have been examined as functional delivery carriers for treatment of diverse diseases (Yamada et al., 2003; Li et al., 2018). Among them, high-density lipoproteins (HDLs) are probably the most extensively studied nanovehicles for drug delivery (Ma et al., 2018; Raut et al., 2018). HDLs are lipid-protein nanoparticles with a mean diameter of 8–10 nm, carrying a large number of different proteins such as apolipoproteins. Whereas HDLs are well known for their role in cholesterol transport and lipid metabolism, increasing evidence has demonstrated their potential for therapy of sepsis (Wu et al., 2004). In addition, both native HDLs and reconstituted HDLs (rHDLs) can function as effective nanoplatforms for targeted drug delivery in cardiovascular and hepatic diseases (Morin et al., 2015; Mo et al., 2016; Ma et al., 2018).

## Applications of Bioinspired Nanoparticles in Antiviral Therapy

It has been demonstrated that antiviral drugs can be loaded in liposomes, micelles, dendrimers, or polymer/lipid nanoparticles to increase their stability, prolong half-life, and realize targeted delivery to specific tissues (such as lymphatic tissues) or cells infected by viruses (Milovanovic et al., 2017; Singh et al., 2017). In this context, albumin nanoparticles loaded with an antiretroviral drug efavirenz were prepared for potential treatment of human immunodeficiency virus (HIV) infection (Jenita et al., 2014), resulting in improved efavirenz delivery to several organs. Also, siRNA nanosomes were formulated by packaging lipid nanoparticles with multiple siRNAs that target the highly conserved 5′-untranslated region of the hepatitis C virus (HCV) genome. In mice, systemic treatment with siRNA nanosomes significantly inhibited HCV replication (Chandra et al., 2012). In addition, intravaginal delivery of siRNA-loaded PLGA nanoparticles effectively prevented genital herpes simplex virus (HSV)-2 infections in mice (Steinbach et al., 2012). By using pathologically responsive nanocarriers, in combination with specific targeting moieties, efficacy of antiviral nanotherapies may be further improved. Studies by Bronshtein et al. showed a biomimetic strategy capable of effectively targeting HIV-infected cells (Bronshtein et al., 2011). In this approach, vesicles were prepared from cells expressing a chemokine receptor CCR5, which is the human receptor for envelope glycoprotein GP120 found on HIV-infected cells. The specific targeting capability of CCR5-conjugated cell-derived vesicles was substantiated by *in vitro* experiments in GP120-expressing HIV model cells.

**Figure 4:**
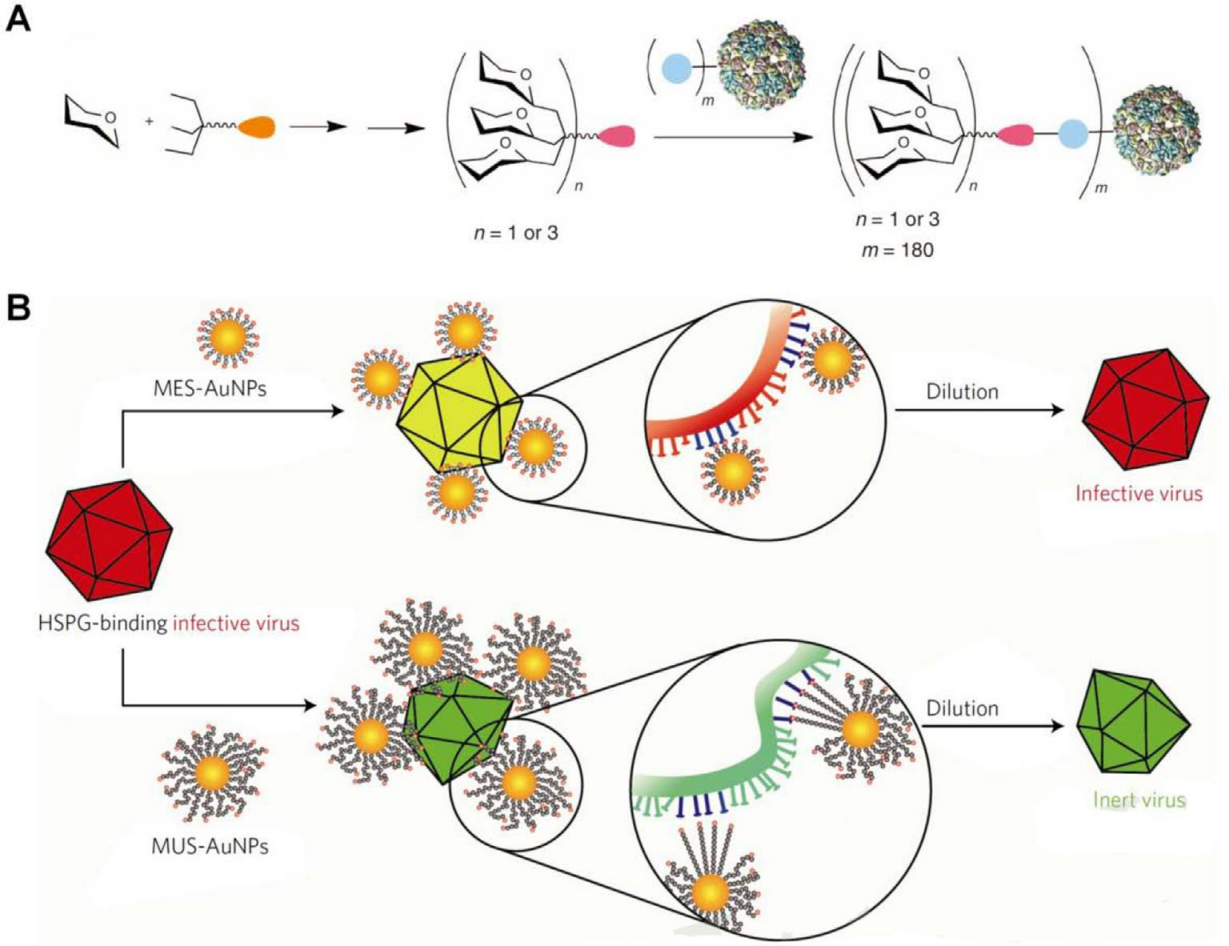
Design of biomimetic antiviral nanotherapies. **(A)** Nested symmetrical assembly of virus-like glycodendrinanoparticles using a tag-and-modify strategy. Glycodendrons are created through iterative multivalent assembly and then attached to multiple tags, each in a monomer protein. **(B)** Cartoon of the virucidal activity of broad-spectrum antiviral nanoparticles MUS : OT-NPs compared to MES-NPs. HSPG, heparin sulfate proteoglycans; MES, 3-mercaptoethylsulfonate; MUS, undecanesulfonic acid; OT, 1-octanethiol. Image **(A)** is reprinted with permission from Ribeiro-Viana et al. (2012). © (2012) Macmillan Publishers Limited. Image **(B)** is reprinted with permission from Cagno et al. (2018). © (2018) Macmillan Publishers Limited.

On the other hand, Davis’ group developed virus-like glycodendrinanoparticles bearing approximately 1,620 glycans (**Figure 4A**) (Ribeiro-Viana et al., 2012), considering the pivotal role of ligand polyvalency in mediating host–pathogen interactions. The constructed nanoparticles, with mean diameter of 32 nm, can mimic viral pathogens with respect to both size and glycosylated surface. In an Ebola viral infection model in mammalian T lymphocytes, these biomimetic glycodendriprotein nanoparticles showed exciting antiviral activity, and they effectively prevented mammalian cell infection by competitively blocking the dendritic cell-specific ICAM-3-grabbing nonintegrin receptor. Based on the similar principle, antiviral nanoparticles (defined as MUS : OT-NPs) were designed and constructed by coating Au nanoparticles with long and flexible linkers mimicking heparin sulfate proteoglycans (HSPG) that are highly conserved receptors for viral attachment (**Figure 4B**) (Cagno et al., 2018). *In vitro* experiments demonstrated that thus developed nanoparticles exhibited desirable virucidal activity in HSPG-dependent viruses, including HSV-1, HSV-2, human papillomavirus (HPV), respiratory syncytial virus (RSV), lentivirus, and dengue virus. Importantly, these broad-spectrum antiviral nanoparticles are active in a mouse model of RSV infection.

## Biomimetic Nanoparticles for Treatment of Bacterial Infections

### Treatment of Bacterial Infections by Toxin-Neutralizing Nanoparticles

Bacteria and fungi generally produce a variety of microbial toxins, classified as endotoxins and exotoxins that can promote infection and disease pathogenesis by directly damaging the host cell membrane and tissues as well as impairing the immune system (Lubran, 1988). Increasing evidence has demonstrated the effectiveness of injectable nanoparticles for biodetoxification by neutralizing toxins (Leroux, 2007; Fang et al., 2015b). For example, synthetic nanoparticles based on copolymers of acrylic acid, N-tert-butylacrylamide, N-isopropylacrylamide, and N,N′-methylenebisacrylamide could effectively capture and neutralize the toxicity of a peptide toxin melittin by electrostatic and hydrophobic interactions (Hoshino et al., 2012; Yoshimatsu et al., 2015). By coating monosialotetrahexosylganglioside (GM1), a key host receptor for cholera toxin, on the surface of PLGA nanoparticles, an effective toxin-neutralizing nanoplatform was obtained, which can selectively neutralize the effects of cholera toxin on epithelial cells (Das et al., 2018). Moreover, the GM1-coated nanoparticle decoys effectively attenuated intestinal secretory responses of live *V. cholerae* in a murine infection model. For these nanoparticles, however, their affinity to different types of toxins and the antivirulence activity remain to be enhanced. In addition, *in vivo* safety of the toxin-affinity nanoparticles derived from acryl compounds should be carefully examined, since they are nondegradable under physiological conditions.

To address these limitations, lipid-based cell membrane-mimicking nanostructures have been applied as novel decoys to efficiently neutralize toxins (Bricarello et al., 2012). Given the fact that the plasmalemma is mainly composed of glycerophospholipids, sphingolipids, and cholesterol, Henry et al. developed liposomes composed of sphingomyelin and high concentrations of cholesterol, which could efficiently absorb membrane-damaging toxins and α-hemolysin (Henry et al., 2015). Furthermore, these artificial liposomes are able to efficiently sequester a broad spectrum of toxins released by a variety of staphylococcal and streptococcal pathogens. Intravenous administration of these exotoxin-absorbing liposomes significantly rescued mice from septicemia caused by *S. aureus* and *S. pneumonia*. Also, liposomes protected mice against invasive pneumonia after intranasal infection with *S. pneumoniae* serotype 2 (strain D39). These beneficial effects were demonstrated to be contributed by sequestration of secreted bacterial toxins, which protected host cells from the lysis and uncontrolled inflammatory responses. Of note, *in vivo* efficacy of liposomes can be further enhanced by combination with antibiotic treatment, as exemplified by using a model antibiotic vancomycin.

Due to the presence of toxin receptors, natural lipoproteins are able to spontaneously interact with different membrane-active toxins such as hemolysins (Badin and Barillec, 1970; Watson et al., 1972; Seganti et al., 1980; Kudo et al., 2001), thereby abrogating their lytic effects. *In vivo* studies in humans demonstrated that HDLs can attenuate inflammatory and coagulation responses after an endotoxin challenge (Birjmohun et al., 2007). To further enhance toxin affinity, ganglioside GM1 was incorporated into rHDLs. Thus, engineered rHDLs showed notably increased binding affinity to cholera toxin, and they effectively reduced toxin attachment to epithelial cells *in vitro* (Bricarello et al., 2010).

**Figure 5:**
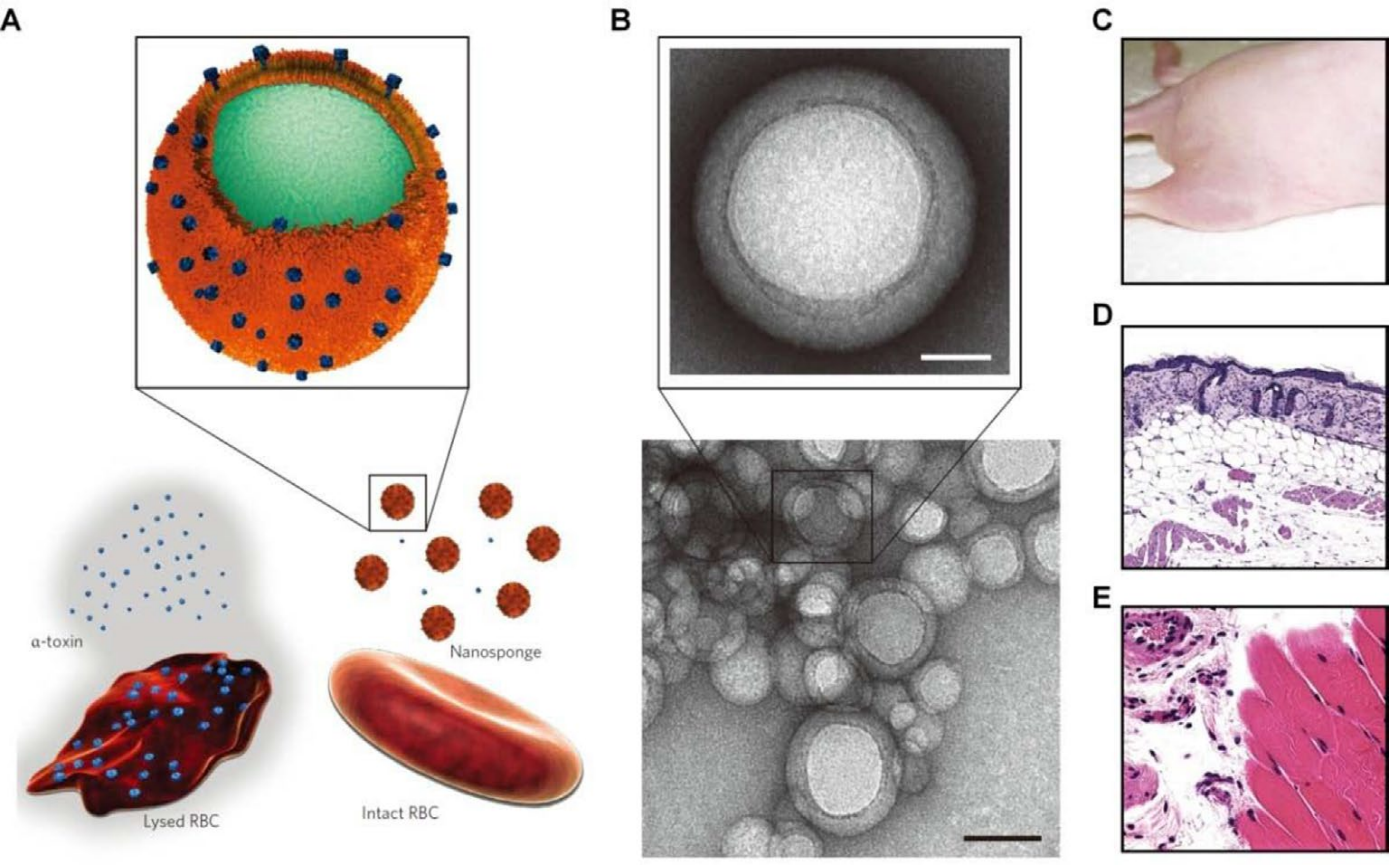
Schematic, characterization, and *in vivo* evaluation of a pore-forming toxin-absorbing biomimetic nanosponge. **(A)** Schematic of a toxin nanosponge and the mechanism of neutralizing pore-forming toxins. The nanosponge consists of substrate-supported RBC bilayer membranes into which pore-forming toxins can be incorporated. **(B)** TEM images of nanosponges mixed with α-toxin (scale bar, 80 nm) and the zoomed-in view of a single toxin-absorbed nanosponge (scale bar, 20 nm). **(C–E)** Mice injected with α -toxin/nanosponges. No skin lesion occurred **(C)**. No abnormality was observed in the epidermis **(D)**. Normal muscle structure was observed **(E)**. Images are reprinted with permission from (Hu et al., 2013a). © (2013) Macmillan Publishers Limited.

Recent studies by Zhang’s group demonstrated that cell membrane-functionalized nanostructures may serve as promising toxin-neutralizing decoys (Fang et al., 2015b). Nanosponges based on RBC membrane-coated PLGA nanoparticles effectively neutralized different pore-forming toxins, such as melittin, α-hemolysin, listeriolysin O, and streptolysin O, and therefore, they could significantly inhibit toxin-induced hemolysis (**Figure 5**) (Hu et al., 2013a; Escajadillo et al., 2017; Zhang et al., 2017b; Chen et al., 2018). Also, the biomimetic nanosponges exhibited desirable neutralization capacity for the whole secreted proteins of methicillin-resistant *S. aureus* (MRSA) (Chen et al., 2019). Furthermore, the antivirulence efficacy of these nanosponges was demonstrated by *in vivo* studies in different mouse models. Through the similar strategy, macrophage-like nanoparticles were developed by wrapping PLGA cores with the macrophage cell membrane (Thamphiwatana et al., 2017). The obtained macrophage-mimetic nanoparticles showed effective binding and neutralization of endotoxins. In a mouse model of *E. coli*-induced bacteremia, treatment with these biomimetic nanoparticles afforded a significant advantage for sepsis control. In addition to lipopolysaccharide (LPS) neutralization, simultaneous sequestration of proinflammatory cytokines was considered to be responsible for the therapeutic benefits of these macrophage-mimicking nanoparticles.

### Treatment of Bacterial Infections by Drug-Loaded Biomimetic Nanoparticles

The combination of nanotechnology with antibiotics represents a very promising strategy for antibacterial therapy. During the past decades, different antibiotics have been loaded in nanoparticles with varied biophysicochemical properties to achieve site-specific and/or intracellular delivery of therapeutics, in particular for combating multi-drug resistant bacteria (Xiong et al., 2014; Zaidi et al., 2017; Gao et al., 2018). In these antibacterial nanotherapies, antibiotics of diverse structures can be physically absorbed on, entrapped in, or covalently conjugated with metal, inorganic, and polymer nanoparticles as well as nanogels, nanoemulsions, liposomes, and hybrid nanovehicles (Pelgrift and Friedman, 2013; Ladaviere and Gref, 2015; Shimanovich and Gedanken, 2016). Also, different antibacterial agents can be co-delivered through specific nanocarriers to achieve synergistic effects. In addition to diffusion, release of antibacterial cargoes may be triggered by pathological signals at infection sites, such as pH, reactive oxygen species, and bacterial toxins or lipases (Ladaviere and Gref, 2015; Moorcroft et al., 2018). Compared to free antibacterial agents, drug-loaded antibiotic nanoparticles displayed enhanced drug stability, targeted delivery, prolonged retention, sustained or responsive release, and enhanced penetrating capability. Due to these multiple functions, antibacterial nanotherapies can effectively overcome decreased uptake and increased efflux of drugs by bacteria, considerably prevent biofilm formation, and notably combat intracellular bacteria.

Despite extensive studies in this field, there are still tremendous challenges in precisely targeting bacteria or infected cells and delivering antibiotics to subcellular compartments with bacteria. Recent studies suggested that biomimetic nanoparticles are effective vehicles for targeted delivery of antibacterial agents. A study by Sun et al. showed that exosomes enhanced delivery of curcumin, a hydrophobic anti-inflammatory drug to activated monocytes *in vivo* (Sun et al., 2010). In mouse models of LPS-induced septic shock and LPS-induced brain inflammation, curcumin-loaded exosomes exhibited more significant protective effects compared to free drug (Sun et al., 2010; Zhuang et al., 2011). Also, exosomes effectively delivered exogenous siRNA to monocytes and lymphocytes (Telemo et al., 2012). Similarly, an exogenous miRNA-155 mimic was successfully delivered to macrophages by exosomes (Momen-Heravi et al., 2014), thereby significantly inhibiting LPS-induced inflammation. Besides exosomes, nanovesicles derived from neutrophil-like cells (i.e., HL-60 cells) were examined for targeted drug delivery. In a mouse model of LPS-induced acute lung inflammation, treatment with HL-60 cell-derived nanovesicles loaded with an anti-inflammatory agent TPCA-1 (an NF-κB inhibitor) significantly inhibited neutrophil infiltration and expression of inflammatory cytokines, by targeting inflamed lung vasculature (Gao et al., 2016). In another study by the same group, intravenous administration of piceatannol-loaded nanovesicles based on HL-60 cells dramatically reduced local and systemic inflammation in LPS-induced mouse models of acute lung injury and sepsis, respectively (Gao et al., 2017).

As well documented, bacteria and other microorganisms can stimulate platelet activation, and the activated platelets may directly bind and capture bacteria (Fitzgerald et al., 2006; Semple et al., 2011). This inherent bacterial adherence capacity of platelets was exploited for targeted delivery of an antibiotic agent vancomycin by using platelet membrane-cloaked PLGA nanoparticles (PNPs) (Hu et al., 2015). Preferential binding of a strain of MRSA252 by PNPs was demonstrated by *in vitro* experiments. Furthermore, in a mouse model of systemic MRSA252 infection, vancomycin-loaded PNPs exhibited significantly better antimicrobial efficacy compared to free drug. In another case, an RBC membrane-coated, redox-responsive nanogel (RBC-nanogel) was examined as a smart delivery nanoplatform (Zhang et al., 2017b). Using vancomycin as a model drug, *in vitro* responsive release was confirmed in reducing environment. Further, the intracellular antibacterial efficacy of vancomycin-loaded RBC-nanogel was substantiated in an *in vitro* model of MRSA USA300-infected macrophages derived from human THP-1 monocytes. Based on a similar design principle, the same group developed antibiotic-loaded nanoparticles coated with plasma membranes of gastric epithelial cells to obtain targeting nanotherapies for the treatment of *H. pylori* infection (Angsantikul et al., 2018). In a mouse model of *H. pylori* infection, the biomimetic nanoparticles containing an antibiotic clarithromycin showed superior therapeutic efficacy compared to free drug and a non-targeting nanoparticle control. Given that host–pathogen adhesion is a common biological event in the pathogenesis of pathogenic infections, this cell membrane-coating nanotherapeutic strategy may serve as a versatile delivery platform to treat numerous infectious diseases.

## Vaccines Based on Bioinspired Nanoparticles

### Virosome-Based Nanovaccines

In addition to delivery of drugs with different structures, virosomes have been extensively studied as a vaccine platform due to their capacity to simultaneously deliver antigens and adjuvants within a single nanoparticle, thereby activating both humoral and cellular immune responses.

RSV may cause infections in the lungs and respiratory tract, leading to severe respiratory diseases in children and the elderly, while currently no effective vaccines are available in the clinic. Stegmann and colleagues prepared lipopeptide P3CSK4-adjuvanted virosomes from RSV envelopes that can induce high virus-neutralizing antibodies after immunization in mice (Stegmann et al., 2010). Moreover, sterilizing immunity to RSV challenge was achieved by these adjuvanted virosomes in mice and cotton rats. Most recently, Lederhofer et al. developed a virosomal RSV vaccine by incorporation of a lipid adjuvant 3-deacyl-phosphorylated hexa-acyl disaccharide (3D-PHAD) in viral membranes containing glycoproteins G and F (Lederhofer et al., 2018). *In vivo* immunization experiments in mice revealed effective production of RSV-specific neutralizing antibodies.

Also, intensive studies have been conducted for the development of influenza vaccines based on virosomes, since influenza virus-caused public health threats and economic damages are still main global concerns. A new chimeric influenza virosome derived from A/PR/8/1934 (H1N1) (PR8) and A/X/47 (H3N2) (X47) viruses was found able to provide complete protection against lethal challenge with PR8 and X47 viruses in mice (Abdoli et al., 2014), by inducing high IgG antibody responses and hemagglutination inhibition (HAI) titers even comparable to those generated by the whole inactivated influenza vaccine. To develop influenza vaccines, a conserved human HLA-A2.1 influenza M1_58–66_ peptide was loaded into virosomes prepared from egg-derived influenza A/PR8/34 H1N1 virus (Soema et al., 2015). The peptide-loaded virosomes induced specific CD8^+^ T cells in HLA-A2 transgenic mice, and the efficacy was further increased by adjuvanting with CpG. To modulate adaptive immune responses in the respiratory tract, Blom et al. designed influenza-derived virosomes loaded with a model antigen ovalbumin (Blom et al., 2017a; Blom et al., 2017b). By presentation with macrophages and dendritic cells after intranasal administration, robust and antigen-specific CD4^+^ T cell proliferation was induced by ovalbumin-containing virosomes, demonstrating their potential for vaccination or immunotherapy of pulmonary diseases such as allergic asthma. In contrast, ovalbumin-loaded liposomes failed to generate a specific T cell activation.

It is worth noting that the efficacy of DNA vaccines can be improved by targeted delivery with virosomes containing influenza virus HA (Gargett et al., 2014). In view of the success of the previously developed virosomal hepatitis A vaccine (i.e., Epaxal), which showed a real-time protection for at least 5.5 years in children (Van Herck et al., 2015), the currently studied virosomal vaccines deserve further intensive development.

### VLP-Based Vaccines

Due to their unique structural features and advantages in safe production, short development time, and many available expression systems (Yan et al., 2015), VLPs are attractive candidates of prophylactic and therapeutic vaccines (Liu et al., 2016). Some VLP-based vaccines, such as hepatitis B and HPV-based VLPs, have been approved for clinical use, and there are still a number of this type of vaccines that are undergoing clinical trials (Huang et al., 2017). Besides their inherent immunogenicity, VLPs can be used as effective platforms for presentation of exogenous antigens in highly immunogenic and multivalent forms, thereby affording chimeric vaccines (Masavuli et al., 2017; Hill et al., 2018). Additionally, therapeutic vaccines have been developed based on VLPs derived from various viruses by presenting self-antigens to treat chronic inflammatory or infectious diseases (Liu et al., 2016; Huang et al., 2017).

In addition to vaccines against viral and bacterial infections, hepatitis B-derived VLPs containing the C-terminus and central repeat region of the circumsporozoite protein (CSP) (i.e., RTS,S) have been successfully developed as a malaria vaccine. Of note, the RTS,S-based vaccine is safe, well tolerated, and immunogenic. It can confer partial efficacy in malaria-naive and malaria-experienced adults as well as children (Regules et al., 2011). Currently, further studies are undergoing to improve efficacy of this type of anti-malarial vaccines, such as by formulating VLPs containing a much higher proportion of CSP (Collins et al., 2017).

### Self-Assembling Nanoparticles

Recently, self-assembling nanoparticles bearing antigens have been explored as novel vaccines against different viral infections (Gallagher et al., 2018; Rappuoli and Serruto, 2019). Studies by Nabel’s group demonstrated that nanoparticles self-assembled by ferritin genetically fused with viral HA can elicit broader and more potent immunity compared to traditional influenza vaccines (**Figure 2A and B**) (Kanekiyo et al., 2013), showing HAI antibody titers >10-fold higher than those achieved by the commercial vaccine. Furthermore, antibodies induced by HA nanoparticles derived from 1999 NC effectively neutralized H1N1 viruses from 1934 to 2007. Immunization with the HA nanoparticle (1999 NC) vaccine protected ferrets challenged with 2007 Brisbane virus. A similar strategy was employed by Corbett and coworkers to design Group 2 influenza virus HA stem antigens that were also displayed on self-assembling ferritin nanoparticles (Corbett et al., 2019). In addition to producing broadly neutralizing antibodies, immunization with these Group 2 HA stem antigen-ferritin nanoparticles protected mice against homosubtypic lethal challenges with influenza viruses. Most recently, a computationally designed, fully synthetic nanoparticle displaying 20 DS-Cav1 trimers of RSV was developed (**Figure 2C–E**) (Marcandalli et al., 2019), which induced neutralizing antibody responses approximately 10-fold higher than trimeric DS-Cav1 in mice and nonhuman primates.

### Vaccines Derived From Other Virus-Mimetic Nanoparticles

Besides the above-mentioned virus-mimicking vaccination approaches, other strategies have been examined for the development of new vaccines. For example, recombinant HA from influenza virus was incorporated into HDL-like nanodisc particles, consisting of lipid bilayers encircled by membrane scaffold proteins (Bhattacharya et al., 2010). Intranasal vaccination with this HA-loaded nanodisc elicited a strong anti-HA IgA response, conferring protection against challenge with the influenza (H1N1) virus, with efficacy comparable to that of the commercial vaccines Fluzone and FluMist. In a recent study, a polymer-templated protein nanoball was constructed, displaying HA1 on its surface with controlled orientation and density (Lee et al., 2018). Vaccination with this virus-mimetic polymer nanoparticle protected mice from H1N1 challenge, in the absence of adjuvant. More importantly, cross-activities against different types of H1N1 influenza viruses were also achieved.

Robinson’s group engineered nanoparticles (20–25 nm) by self-assembly of synthetic coiled-coil lipopeptides (Ghasparian et al., 2011), on which helper T-cell and B-cell epitopes can be displayed by linking to each lipopeptide chain. In mice and rabbits, the obtained nanoparticles afforded strong humoral immune responses. Based on a similar design principle (Riedel et al., 2011), an antibody F425-B4e8-bound V3 loop (from GP120 of HIV-1) mimic was coupled to lipopeptide nanoparticles. The obtained nanoparticles could elicit antibodies and block infection by some strains of HIV-1 in rabbits.

### Vaccines Derived From Bacterial Extracellular Membrane Vesicles

Due to their immunogenic properties, self-adjuvant capacity, potential for modification by genetic engineering, cellular uptake ability by antigen-presenting cells, and the capability to present exogenous antigens, bacterial extracellular vesicles have received increasing attention in vaccine development (Yu et al., 2018). Since an OMV-based vaccine against serogroup B *N. meningitidis* was successfully used for epidemic control in several countries in 2004 (with vaccination efficacies varying from 83% to 94%) (Danzig, 2004), either natural or bioengineered OMVs have been intensively investigated to develop new vaccines with enhanced efficacy against meningococcal disease.

By genetically adding a factor H binding protein (fHBP, a desirable antigen) into trivalent native OMVs derived from *N. meningitidis* serogroup B strains, the immunogenicity and breadth of the original OMV vaccines were notably improved in mice and infant rhesus macaques (Zhang et al., 2016). A recent study by Beernink et al. revealed that a native OMV with genetically attenuated endotoxins and overexpressed fHBP has broader meningococcal bactericidal activity in mice (Granoff et al., 2019). Moreover, this OMV vaccine elicited strong serum bactericidal antibody against gonococci and meningococci. Also, it was found that OMVs displaying a conserved surface polysaccharide antigen poly-N-acetyl-D-glucosamine (PNAG) induced high titers of PNAG-specific IgG antibodies after vaccination in mice (Stevenson et al., 2018). Further, mice immunized with the glycosylated OMVs showed protective immunity against unrelated pathogens, such as *S. aureus* and *Francisella tularensis subspecies Holarctica*. Additionally, extensive studies have been conducted to combat other infectious diseases based on different OMVs derived from *S. typhimurium, A. baumannii, P. gingivalis, T. pallidum, B. burgdorferi, B. pertussis, N. lactamica, H. pylori, P. aeroginosa, V. cholera, Y. pestis*, and *B. melitensis* (Collins, 2011; Unal et al., 2011; Yu et al., 2018). For example, a thermostable spray-dried *B. pertussis* OMV vaccine was developed by Kanojia and coworkers, which displayed desirable immune responses and protection against challenge with live *B. pertussis* after pulmonary immunization (Kanojia et al., 2018). Flagellin-deficient *S. typhimurium* OMVs, in combination with outer membrane proteins, induced high cellular immune responses (Liu et al., 2018a). Also, OMVs enhanced cross-protection on challenge by wild-type virulent *S. choleraesuis* and *S. enteritidis*.

In addition to OMVs derived from Gram-negative bacteria, they can be released by Gram-positive bacteria, such as *S. aureus*, *B. anthracis*, *S. coelicolor*, *L. monocytogenes*, and *B. subtilis* (Lee et al., 2009; Rivera et al., 2010; Brown et al., 2015). A recent study showed that OMVs from community-associated MRSA contain cytosolic, surface, and secreted proteins (Wang et al., 2018b). Moreover, OMVs with genetically engineered, detoxified cytolysins can elicit toxin-neutralizing antibodies in mice, thereby protecting the animals against lethal *S. aureus* sepsis.

### Vaccines Based on Other Cell Membrane-Derived Nanoparticles

Besides vesicles released by different bacteria, nanotechnology has been combined with bacterial mimetic strategies to develop effective vaccine platforms. Recently, a facile approach was established to prepare double-layered membrane vesicles (DMVs) by nitrogen cavitation (Wang et al., 2018a). DMVs based on a model pathogen *P. aeruginosa* exhibit a whole bacterial membrane and contain a variety of membrane proteins. Consistently, immunization with DMVs effectively promoted the innate and adaptive immune responses in a sepsis model of mice induced by *P. aeruginosa*, thereby improving mouse survival.

On the other hand, *E. coli* OMVs were coated on gold nanoparticles, resulting in hybrid, biomimetic nanovaccines (BM-AuNPs) (Gao et al., 2015). After subcutaneous injection of BM-AuNPs in mice, rapid activation and maturation of dendritic cells in the lymph nodes were observed. Of note, BM-AuNPs elicited significantly higher bacteria-specific antibody responses compared to OMVs alone. Furthermore, immunization with BM-AuNPs induced strong Th1- and Th17-biased cell responses against *E. coli* challenge. In another strategy established by the same group (Hu et al., 2013b), staphylococcal α-hemolysin was entrapped in RBC membrane-coated PLGA nanoparticles. Vaccination with the nanoparticle-detained toxin showed superior protective immunity against toxin-mediated adverse effects. Moreover, multiple virulent toxins secreted by an MRSA strain USA300 were concurrently and naturally presented on a nanosponge derived from RBC membrane-coated PLGA nanoparticles (Wei et al., 2017). The formulated multivalent nanotoxoids elicited desirable anti-virulence immunity that can combat live bacterial infections in mice. Notably, both *in vitro* and *in vivo* studies on nanotoxoids revealed good safety profile. Consequently, this biomimetic nanoparticulate platform affords a versatile approach for vaccine development against many infectious diseases.

## Concluding Remarks and Future Perspectives

Biomimetic engineering of nanoparticles is a rapidly developing field, with tremendous advances in the last decade. Taking advantage of the specific delivery and translocation mechanisms adopted by pathogens and mammalian cells, the bioinspired nanoparticles have diverse functions, such as prolonged circulation, enhanced accumulation at infected sites, and reduced off-target effects in healthy tissues. Despite the approval of virosomes and VLPs as novel vaccines, there are still considerable translation barriers for the majority of currently developed biomimetic nanotherapies.

First, biological complexity provides desirable functions to bioinspired nanoplatforms, which simultaneously causes challenges for process control, purification, scaling up production, and reproducible manufacture in the preparation stage. On the basis of the quality-by-design principle, the virtue of simplicity should be followed to facilitate translation studies. Second, regardless of nanoparticles engineered by various biomimetic approaches, their structural features and molecular components are only poorly characterized. Future studies are required to elucidate details regarding the exact components as well as the distribution, arrangement, and orientation of specific biomolecules on the surface of bioinspired nanoparticles, since these parameters are extremely important for their *in vivo* fates and therapeutic efficacies. In this aspect, emerging technologies such as multiplexed protein analysis, proteomics, and imaging mass spectrometry can be used. Establishment of the structure–property correlation will be beneficial for simplifying or optimizing preparation procedures of existing biomimetic nanoparticles. This can also promote the design and development of more effective nanoplatforms. Third, the majority of research works on biomimetic nanoparticles has been focusing on cancer therapy, while only a limited number of studies concern the treatment of infectious diseases, with the exception of virosomes and VLPs. It is worth noting that the biological features of most biomimetic nanoparticles, especially those based on viruses and bacteria, do not guarantee their ideal delivery in cancer treatment. Consequently, the effectiveness and long-term safety issues of newly bioengineered nanotherapies need to be confirmed by comprehensive anti-infective studies. Likewise, mechanistic studies should be conducted to address molecular and cellular events dominating *in vivo* biopharmaceutical and pharmacokinetic profiles of biomimetic nanotherapies currently developed. Finally, it is necessary to further expand the types and functions of biomimetic nanoplatforms, according to new advances in biological and life sciences, in combination with the revolution in nanotechnology. Other cutting-edge technologies, such as computational design, materials genome, and artificial intelligence, can be integrated to discover more effective and translational nanoparticles based on the bioengineering strategies.

Despite the above-mentioned challenges and limitations of bioinspired nanotherapies for anti-infective applications, we may anticipate that the biomimetic strategy-based nanomedicine field will provide novel therapeutics against infectious diseases in the near future, in view of the notable clinical success of bioinspired nanovaccines.

## Author Contributions

GY drafted the manuscript. GY and SC created the figures and performed literature searches. JZ revised the manuscript and edited the final draft.

## Funding

This study was supported by the National Natural Science Foundation of China (no. 81471774) and the Program for Distinguished Young Scholars of TMMU.

## Conflict of Interest Statement

The authors declare that the research was conducted in the absence of any commercial or financial relationships that could be construed as a potential conflict of interest.

## Abbreviations

CDVs, cell-derived vesicles; CSP, circumsporozoite protein; DMVs, double-layered membrane vesicles; 3D-PHAD, 3-deacyl-phosphorylatedhexa-acyl disaccharide; fHBP, factor H binding protein; GM1, monosialotetrahexosylganglioside; HA, hemagglutinin; HAI, hemagglutination inhibition; HBV, hepatitis B virus; HCV, hepatitis C virus; HDLs, high-density lipoproteins; HIV, human immunodeficiency virus; HPV, human papillomavirus (HPV); HSPG, heparin sulfate proteoglycans; HSV, herpes simplex virus; HVJ, hemagglutinating virus of Japan; ICAM, intercellular adhesion molecule; LPS, lipopolysaccharide; MRSA, methicillin-resistant *S. aureus*; NA, neuraminidase; OMVs, outer membrane vesicles; PEG, polyethylene glycol; PLGA, poly(lactic-co-glycolic acid); PNAG, poly-N-acetyl-D-glucosamine; PNPs, platelet membrane-cloaked PLGA nanoparticles; RBCs, red blood cells; rHDLs, reconstituted HDLs; RSV, respiratory syncytial virus; VLPs, virus-like particles; VSV, vesicular stomatitis virus.
